# Contrast-induced encephalopathy—neuroimaging findings and clinical relevance

**DOI:** 10.1007/s00234-022-02930-z

**Published:** 2022-03-15

**Authors:** Frederick J. A. Meijer, Stefan C. A. Steens, Anil M. Tuladhar, Ewoud D. van Dijk, Hieronymus D. Boogaarts

**Affiliations:** 1 Department of Medical Imaging, Radboud University Medical Center, Geert Grooteplein 10, Postbus 9101, 6500 HB, Nijmegen, The Netherlands; 2 Department of Neurology, Donders Center for Medical Neurosciences, Radboud University Medical Center, Nijmegen, The Netherlands; 3 Department of Neurosurgery, Radboud University Medical Center, Nijmegen, the Netherlands

**Keywords:** Brain, CT, Angiography, Contrast, Encephalopathy

## Abstract

Contrast-induced encephalopathy (CIE) is a rare encephalopathic condition after the administration of a contrast agent. The diagnosis of CIE is challenging because of the heterogeneity and non-specificity of the clinical presentation. The clinical course is usually favorable with full recovery within 48–72 h in most patients, although comorbidity is of relevance and contributes to the clinical outcome. It is expected that the incidence of CIE is currently increasing, due to an increase in endovascular and diagnostic imaging procedures using iodinated contrast. It is important to include CIE in the differential diagnosis when patients deteriorate during, or immediately after, contrast administration, even when only a small amount of non-ionic contrast agent is used. When CIE is considered to be the most likely explanation for the clinical symptoms, it is advised to refrain from unnecessary additional contrast studies such as angiography or perfusion CT.

## Introduction

Contrast-induced encephalopathy (CIE) is a rare complication following the intravenous or intra-arterial administration of an iodinated contrast agent. Clinical manifestations include visual disturbances (transient cortical blindness is the most common manifestation), motor or sensory deficits, aphasia, altered consciousness, and seizures [1–3]. Due to the heterogeneity of the clinical presentation and the broad differential diagnosis, neuroimaging has a crucial role in the diagnosis of CIE while excluding other more frequent causes, such as recurrent cerebral ischemia, hemorrhage, and posterior reversible encephalopathy syndrome (PRES). On brain CT, CIE is characterized by hyperdense brain swelling and increased density of the cerebrospinal fluid (CSF) [1–3]. The incidence of CIE ranges between 0.3% and 2%, depending on the patient characteristics and type of procedure [4–6]. The type of contrast agent is also of influence, as an incidence up to 4% has been reported in hyperosmolar, ionic contrast agents, which are nowadays infrequently used [2]. CIE has also been reported in non-ionic, iso-osmolar agents, which are less neurotoxic and have a lower incidence of CIE [1]. It is however expected that the incidence of CIE is currently increasing, because cerebrovascular diseases are more frequently treated with endovascular procedures, e.g., intra-arterial mechanical thrombectomy in acute ischemic stroke, or endovascular treatment of cerebral aneurysms, but also due to an overall increase in diagnostic imaging with contrast administration. Recently, an incidence of 1.7% has been reported in patients with acute ischemic stroke undergoing endovascular treatment [5], although this could still be an underestimation as CIE is not always recognized. The clinical course of CIE is usually favorable with full recovery in most patients within 48–72 h [1–3], but comorbidity is of relevance and contributes to the clinical outcome as illustrated by two case descriptions below.

## Illustrative cases

### Case 1

**Fig. 1:**
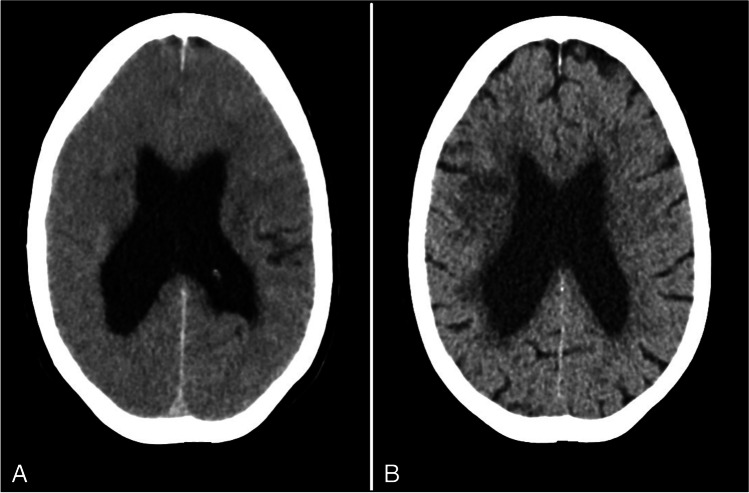
Case 1. Brain swelling on non-contrast CT performed directly after mechanical thrombectomy (**A**), which resolved on follow-up CT 4 days later (**B**)

A 62-year-old female patient presented at the emergency room (ER) because of sudden onset of slurred speech and left hemiparesis. Her medical history noted hypertension, chronic obstructive pulmonary disease, and secondary seizures following intracerebral hemorrhage. There was no impairment of renal function. Brain CT showed no evidence of intracranial hemorrhage, and CT angiography demonstrated an occlusion of the right M1 branch of the middle cerebral artery (MCA). The patient was transferred to the angiography suite, and mechanical thrombectomy was performed successfully. In total, 220 ml non-ionic contrast agent was used for the CT and angiography procedures (Iomeprol 300 mgi/ml). Directly after the procedure, the patient deteriorated with loss of consciousness (Glasgow-coma score 8) and respiratory insufficiency. No seizures were observed. Brain CT was performed, which demonstrated diffuse cerebral swelling (Fig. 1). The patient was transferred to the intensive care unit (ICU) and was intubated. In the following days, the patient improved neurologically and was responsive and orientated. A brain CT performed 4 days after admission demonstrated a small hypodense infarct area in the right MCA territory without any signs of brain swelling. Unfortunately, 3 weeks after admission to the ICU, the patient died due to respiratory failure.

### Case 2

**Fig. 2:**
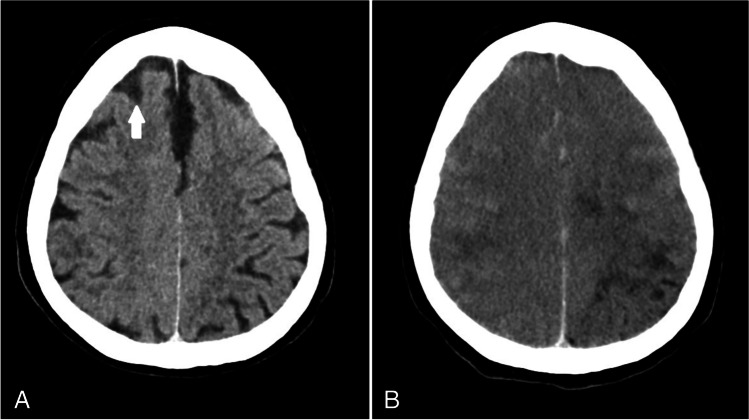
Case 2. Non-contrast brain CT performed directly after percutaneous coronary intervention (**A**) showed increased density of the CSF (arrow). Follow-up CT 7 h later (**B**) demonstrated progressive brain swelling

A 65-year-old female underwent a percutaneous coronary intervention (PCI) procedure for right coronary artery revascularization. Her medical history noted hypertension, type 2 diabetes mellitus, asthmatic bronchitis, and coronary artery disease with unstable angina pectoris. There was no impairment of renal function. For the PCI, 110 ml non-ionic contrast agent was used (Iomeprol 300 mgi/ml). During the PCI procedure, the patient developed seizures and was unresponsive (Glasgow-coma score 6). Head CT was performed directly after the procedure and was repeated 7 h later, which demonstrated progressive swelling of the brain with effacement of the CSF spaces (Fig. 2). In the following days, there was a gradual improvement in consciousness, but the patient suffered from a left hemiparalysis. Brain MRI was performed 10 days after the PCI procedure, which demonstrated parenchymal damage of cortical-subcortical areas in the right hemisphere. There was no typical vascular territory distribution, and the imaging findings were not consistent with recent infarction. One month after admission, the patient still suffered from a left hemiparesis, and she was transferred to a rehabilitation clinic.

## Discussion

Contrast-induced encephalopathy can occur after different types of diagnostic or therapeutic procedures, although direct intra-arterial or intrathecal contrast administration, e.g., in neuro-interventional and cardiovascular procedures, has a higher risk of CIE as compared to intravenous contrast administration and often occurs in the territory of the blood vessels where the contrast agent has been injected [4]. The type and concentration of the contrast agent are of influence, where a larger volume, hyperosmolarity, higher concentrated, and ionic contrast agents are associated with a higher risk of CIE [1–3]. The presumed pathophysiology is direct neurotoxicity of the contrast agent, leaked in the brain parenchyma and subarachnoid space, due blood–brain barrier disruption and endothelial dysfunction [7]. Because CIE can also be seen in non-ionic, iso-osmolar contrast agents, other pathophysiological mechanisms have been proposed including arterial vasospasm with microcirculation disruption [8]. Risk factors of CIE include conditions which compromise the blood–brain barrier, such as hypertension, impaired cerebral autoregulation, and cerebral ischemia, but also diabetes, renal impairment, large contrast volume, previous adverse reaction to iodinated contrast media, and male gender pose a higher risk of CIE [1–5]. It should be emphasized that CIE is considered not to be an immune-mediated allergic response.

The diagnosis of CIE is challenging, especially in acute ischemic stroke where other post-thrombectomy complications should be considered. Proposed diagnostic criteria for CIE after acute ischemic stroke include a clinical deterioration, or delayed improvement, that cannot be explained by the original ischemic area, reperfusion injury, recurrent stroke, or hemorrhagic transformation [5]. The diagnosis of CIE is therefore by exclusion but can be supported by the clinical course and neuro-imaging findings, such as edematous swelling of brain areas beyond the infarct core and contrast staining in the brain parenchyma or subarachnoid space [5]. This can however be subtle or absent in the acute phase, as it is a dynamic process where brain swelling can be progressive and beyond infarcted areas in the hours after contrast injection. In order to differentiate between hemorrhage and contrast agent, dual-energy CT can be used [9]. On MRI, FLAIR hyperintense swollen cortical areas can be seen, and DWI is used to differentiate CIE from acute ischemia (restricted diffusion is seen in the latter but not in the former). In the differential diagnosis, PRES should also be considered, especially when a more or less symmetrical pattern of cortical and subcortical abnormalities is observed. There is indeed a possible overlap in the pathophysiology of CIE and PRES [10].

The clinical course of CIE is usually beneficial with transient symptoms, and most cases improve within 48–72 h. There is little evidence about the optimal treatment, but close observation is advised, and intravenous fluid administration can be considered. Sometimes, anticonvulsive treatment is needed. In severe cases, it has been reported that mannitol could be considered to reduce cerebral edema; the use of anti-inflammatory drugs, e.g., steroids, is however controversial [1, 2]. Although clinical symptoms can be mild with a usually favorable clinical course, brain tissue damage and persistence of neurological deficits can occur as a result of CIE. Comorbidity is of relevance, and the clinical course can be serious with potential fatality. Therefore, it is important to include CIE in the differential diagnosis when acute neurological symptoms occur during, or immediately after, examinations with contrast administration, even when only a small amount of a non-ionic contrast agent is used. When CIE is considered to be the most likely cause for the clinical symptoms, it is advised to refrain from additional unnecessary contrast studies, such as angiography or perfusion CT.

## Data Availability

Not applicable.
